# Laparoscopic Box Training with Four Different Modules in a Tertiary Education and Research Hospital, Ankara, Turkey

**DOI:** 10.1055/s-0039-1688411

**Published:** 2019-04-24

**Authors:** Omer L. Tapisiz, Sadiman Kiykac Altinbas, Ozlem Moraloglu Tekin

**Affiliations:** 1 Department of Obstetrics and Gynecology, University of Health Sciences, Etlik Zubeyde Hanim Women's Health Training and Research Hospital, Ankara, Turkey

**Fig. 1:**
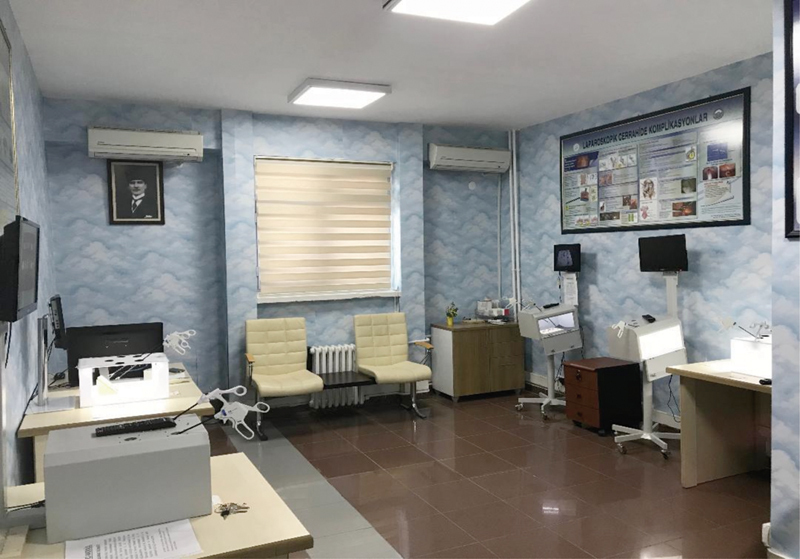
View of the laparoscopic training room.

Dear Editor,

Laparoscopic surgery (LS) has had a fundamental role in gynecology over the past 2 decades. Because LS is now widely accepted, training residents to perform laparoscopic procedures is essential.^1^ Therefore, simultaneously, the interest in training programs to teach technical skills is gaining ground rapidly.^2^ ^3^ Laparoscopic surgery has obtained a major position within surgical specialties. For LS, additional psychomotor and hand-eye coordination skills are needed.^4^ To learn these skills, effective preclinical simple box trainers have been developed. In the light of these data, we have created a well-designed LS training room (Fig. 1), and arranged four different modules. Here, we would like to present the LS training room and box trainers of our hospital, and also indicate the importance of these training activities in the education of the residents.

**Fig. 2:**
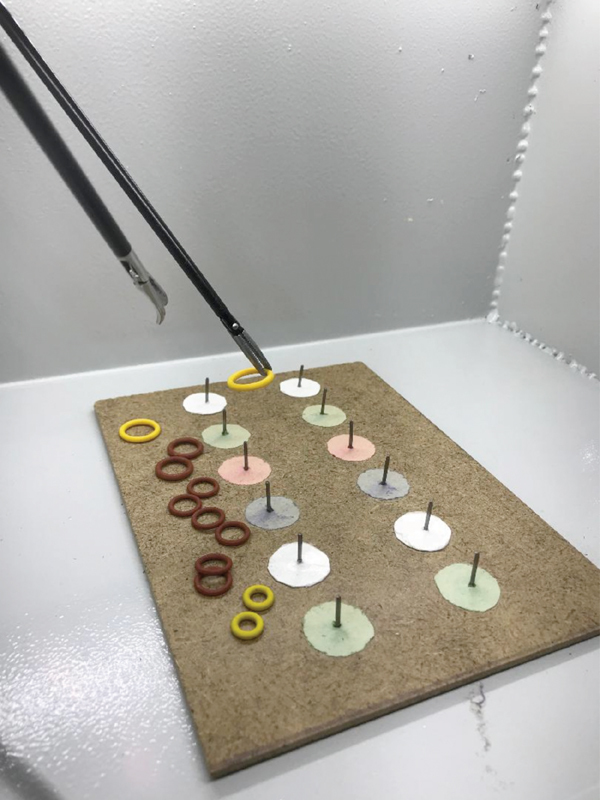
Training module 1.

In Fig. 2, the training module (TM) 1 is presented. In this TM, the surgeon should attach the rings to the nails while paying attention to their colors and sizes. In this process, the surgeon should also change the rings between the right and left hands.

**Fig. 3:**
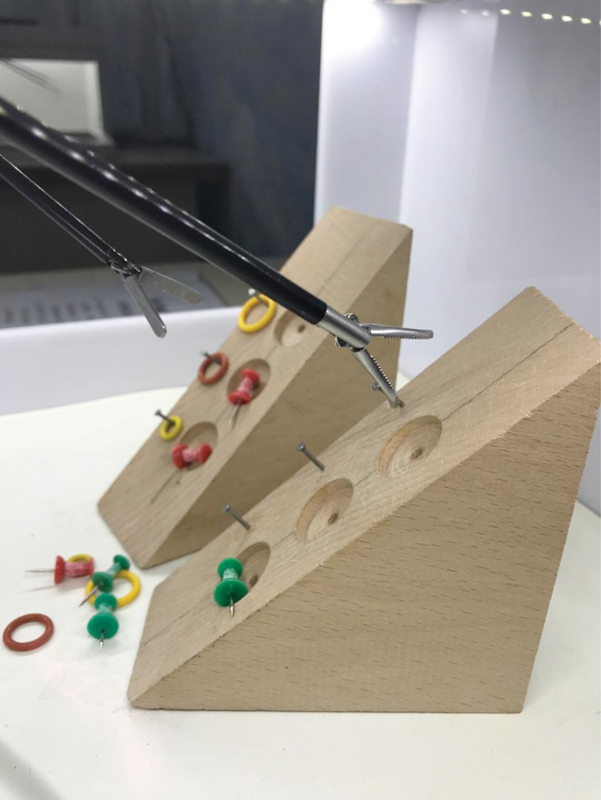
Training module 2.

In Fig. 3, the TM 2 is presented. In this TM, the surgeon should place the pins in wooden hollows while paying attention to their colors. In this process, the surgeon should first pick the pins up from their color parts, then change them between the right and left hands by holding the needle part.

**Fig. 4:**
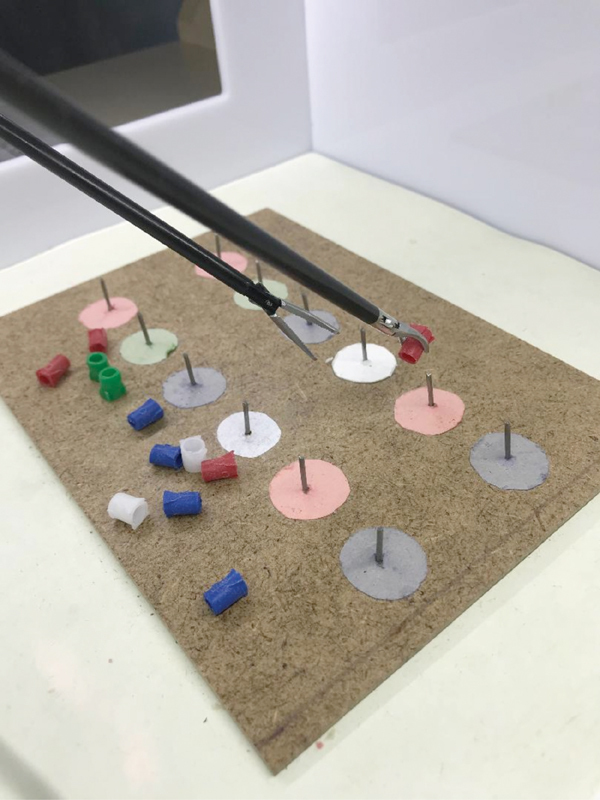
Training module 3.

In Fig. 4, the TM 3 is presented. In this TM, the surgeon should attach the small plastic pipes to the nails while paying attention to their colors.

**Fig. 5:**
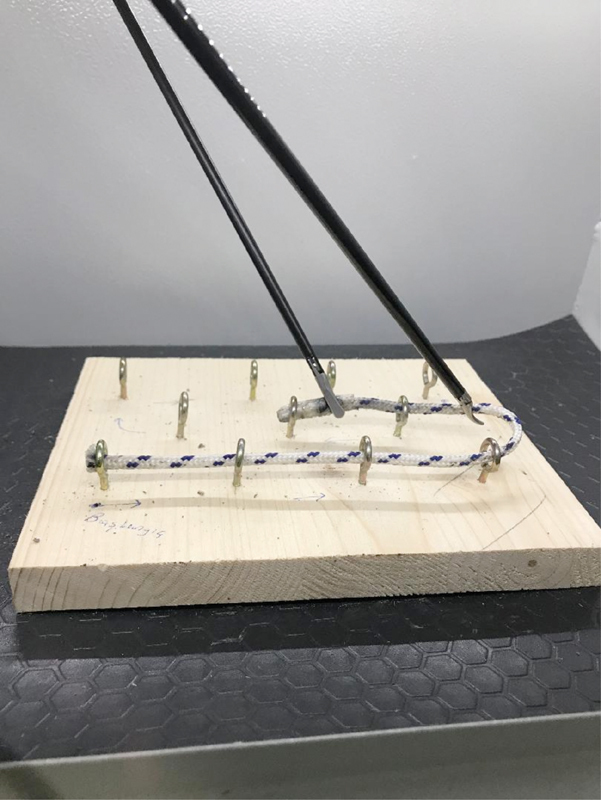
Training module 4.

In Fig. 5, the TM 4 is presented. In this TM, the surgeon should pass the rope through the metal rings with the help of both hands. The direction should be from left to right and down/up to up/down.

In our opinion, for the development of effective and complete laparoscopic training programs, these integrated modules can be a practical answer. All these TMs improve the laparoscopic skills, and training with a virtual reality simulator or box trainer should be considered before actual laparoscopic procedures are performed. Therefore, it should be kept in mind that laparoscopic training hospitals should coordinate a laparoscopic training room that includes a traditional box trainer or with both box trainers and a virtual reality simulator.
